# Correction to: Regression of solid breast tumours in mice by Newcastle disease virus is associated with production of apoptosis related-cytokines

**DOI:** 10.1186/s12885-019-5588-2

**Published:** 2019-04-24

**Authors:** Juraimi Raihan, Umar Ahmad, Yoke Keong Yong, Zolkapli Eshak, Fauziah Othman, Aini Ideris

**Affiliations:** 1 Department of Human Anatomy, Faculty of Medicine and Health Sciences, Universiti Putra Malaysia, UPM, 43400 Serdang, Selangor Malaysia; 2 Ministry of Health Malaysia, 62590 Putrajaya, Malaysia; 3 Medical Genetics Laboratory, Genetics and Regenerative Medicine Research Centre, Faculty of Medicine and Health Sciences, University Putra Malaysia, 43400 Serdang, Selangor Malaysia; 4 Genetics and Cytogenetics Unit, Department of Anatomy, Faculty of Medicine, Bauchi State University, Itas/Gadau, PMB 65 Nigeria; 5 Faculty of Pharmacy, Universiti Teknologi Mara, 42300 Bandar Puncak Alam, Selangor Malaysia; 6 Faculty of Veterinary Medicine, Universiti Putra Malaysia, 43400 Serdang, Selangor Malaysia

**Correction to: Raihan et al. BMC Cancer (2019) 19:315.**

**https://doi.org/10.1186/s12885-019-5516-5**

Following publication of the original article [1], it was reported that the last sentence of the *Conclusion* section was incorrect. The sentence should read as follows:

“However, further experimental studies need to be carry out in vitro and in vivo to decipher the molecular mechanism underlaying tumour cytolysis and cytokines released due to NDV infection”.

The original article [1] has been corrected. The publishers apologise for any inconvenience caused.
