# Supplementary material for: Regression of solid breast tumours in mice by Newcastle disease virus is associated with production of apoptosis related-cytokines
Source: BMC Cancer. 2019 Apr 4;19:315. doi: 10.1186/s12885-019-5516-5 (PMC6449948; doi:10.1186/s12885-019-5516-5)
Supplement: Supplementary file 1 — Table S1. Concentration of IL-6 in both the NDV treated and control groups expressed in pg/ml throughout week 1 to week 4. (DOCX 15 kb) [file 12885_2019_5516_MOESM1_ESM.docx]

**Table S1:**

| **Groups/Week** | **Week 1** | **Week 2** | **Week 3** | **Week 4** |
| --- | --- | --- | --- | --- |
| **NC** | 1.3 ± 0.8 | 8.2 ± 0.2 | 9.5 ± 0.9 | 9.5 ± 1.2 |
| **CC** | 18.4 ± 0.1^a^ | 33.4 ± 4.6^a^ | 54.2 ± 3.9^a^ | 54.2 ± 8.7^a^ |
| **CT** | 15.5 ± 1.3^b^ | 21.6 ± 0.5^b^ | 33.3 ± 5.3^b^ | 38.8 ± 0.4^b^ |
| **NDV8** | 3.5 ± 0.4^b^ | 4.1 ± 0.3^b^ | 5.2 ± 0.3^b^ | 5.2 ± 0.1^b^ |
| **NDV16** | 3.0 ± 0.1^b^ | 3.6 ± 0.5^b^ | 5.3 ± 0.2^b^ | 4.5 ± 0.3^b^ |
| **NDV32** | 2.5 ± 0.2^b^ | 2.8 ± 0.1^b^ | 6.6 ± 0.2^b^ | 5.7 ± 0.1^b^ |
| **NDV64** | 2.1 ± 0.1^b^ | 2.0 ± 0.1^b^ | 4.1 ± 0.1^b^ | 5.4 ± 0.5^b^ |
| **CNDV8** | 3.7 ± 0.2^b^ | 3.5 ± 0.1^b^ | 3.2 ± 0.3^b^ | 6.5 ± 0.2^b^ |
| **CNDV16** | 14.2 ± 0.1^b^ | 13.6 ± 0.5^b^ | 11.9 ± 0.2^b^ | 9.3 ± 0.3^b^ |
| **CNDV32** | 6.5 ± 0.2^b^ | 20.1 ± 0.7^b^ | 52.5 ± 5.6 | 46.0 ± 0.9^b^ |
| **CNDV64** | 9.5 ± 0.1^b^ | 12.5 ± 0.4^b^ | 14.7 ± 0.3^b^ | 16.7 ± 1.3^b^ |
| **CNDV8+T** | 8.0 ± 0.8^b^ | 12.6 ± 0.2^b^ | 40.1 ± 6.2^b^ | 38.6 ± 0.1^b^ |
| **CNDV16+T** | 2.9 ± 0.2^b^ | 10.3 ± 0.2^b^ | 35.8 ± 6.5^b^ | 32.9 ± 0.9^b^ |
| **CNDV32+T** | 5.0 ± 0.1^b^ | 7.6 ± 0.2^b^ | 12.7 ± 0.4^b^ | 19.0 ± 1.0^b^ |
| **CNDV64+T** | 9.4 ± 0.2^b^ | 13.5 ± 0.5^b^ | 13.9 ± 1.4^b^ | 15.0 ± 2.9^b^ |
