# Supplementary material for: Regression of solid breast tumours in mice by Newcastle disease virus is associated with production of apoptosis related-cytokines
Source: BMC Cancer. 2019 Apr 4;19:315. doi: 10.1186/s12885-019-5516-5 (PMC6449948; doi:10.1186/s12885-019-5516-5)
Supplement: Supplementary file 2 — Table S2. Concentration of IFN-γ in both the NDV treated and control groups expressed in pg/ml throughout week 1 to week 4. (DOCX 15 kb) [file 12885_2019_5516_MOESM2_ESM.docx]

**Table S2:**

| **Groups/Week** | **Week 1** | **Week 2** | **Week 3** | **Week 4** |
| --- | --- | --- | --- | --- |
| **NC** | 4.2 ± 0.7 | 2.3 ± 0.3 | 3.5 ± 0.5 | 4.1 ± 0.1 |
| **CC** | 13.1 ± 0.1^a^ | 33.6 ± 4.5^a^ | 43.3 ± 11.6^a^ | 40.1 ± 2.4^a^ |
| **CT** | 23.0 ± 0.8^b^ | 25.5 ± 0.5^b^ | 30.9 ± 0.7^b^ | 32.0 ± 0.4^b^ |
| **NDV8** | 29.0 ± 0.5^b^ | 27.5 ± 0.9^b^ | 23.4 ± 0.7^b^ | 25.7 ± 0.5^b^ |
| **NDV16** | 24.1 ± 0.9^b^ | 25.2 ± 1.9^b^ | 25.1 ± 0.8^b^ | 23.3 ± 0.3^b^ |
| **NDV32** | 34.5 ± 0.2^b^ | 26.4 ± 1.5^b^ | 23.9 ± 1.2^b^ | 21.4 ± 0.1^b^ |
| **NDV64** | 28.7 ± 1.2^b^ | 23.1 ± 0.2^b^ | 24.5 ± 2.6^b^ | 19.1 ± 0.1^b^ |
| **CNDV8** | 39.4 ± 0.5^b^ | 23.4 ± 0.2^b^ | 21.9 ± 0.6^b^ | 12.3 ± 0.1^b^ |
| **CNDV16** | 44.5 ± 1.4^b^ | 30.7 ± 0.6^b^ | 22.6 ± 1.7^b^ | 19.1 ± 0.1^b^ |
| **CNDV32** | 27.0 ± 0.9^b^ | 25.0 ± 0.1^b^ | 22.4 ± 0.1^b^ | 21.4 ± 0.1^b^ |
| **CNDV64** | 43.7 ± 0.4^b^ | 32.3 ± 0.5 | 29.3 ± 0.2^b^ | 25.3 ± 3.3^b^ |
| **CNDV8+T** | 32.5 ± 0.2^b^ | 14.9 ± 1.2^b^ | 12.1 ± 1.0^b^ | 10.2 ± 0.1^b^ |
| **CNDV16+T** | 32.0 ± 0.2^b^ | 22.3 ± 0.9^b^ | 13.3 ± 0.9^b^ | 11.3 ± 1.3^b^ |
| **CNDV32+T** | 40.4 ± 1.3^b^ | 37.7 ± 2.1^b^ | 19.6 ± 2.5^b^ | 18.2 ± 1.1^b^ |
| **CNDV64+T** | 43.8 ± 0.1^b^ | 39.1 ± 0.6^b^ | 29.2 ± 4.1^b^ | 25.3 ± 1.3^b^ |
