# Supplementary material for: Regression of solid breast tumours in mice by Newcastle disease virus is associated with production of apoptosis related-cytokines
Source: BMC Cancer. 2019 Apr 4;19:315. doi: 10.1186/s12885-019-5516-5 (PMC6449948; doi:10.1186/s12885-019-5516-5)
Supplement: Supplementary file 3 — Table S3. Concentration of MCP-1 in both the NDV treated and control groups expressed in pg/ml throughout week 1 to week 4. (DOCX 15 kb) [file 12885_2019_5516_MOESM3_ESM.docx]

**Table S3:**

| **Groups/Week** | **Week 1** | **Week 2** | **Week 3** | **Week 4** |
| --- | --- | --- | --- | --- |
| **NC** | 0.6 ± 0.1 | 1.3 ± 0.3 | 2.1 ± 0.4 | 0.8 ± 0.1 |
| **CC** | 5.0 ± 0.9^a^ | 4.8 ± 0.9^a^ | 7.4 ± 1.0^a^ | 11.2 ± 2.9^a^ |
| **CT** | 1.5 ± 0.1^b^ | 1.4 ± 0.3^b^ | 1.5 ± 0.2^b^ | 1.7 ± 0.2^b^ |
| **NDV8** | 0.0 ± 0.0^b^ | 0.0 ± 0.0^b^ | 0.0 ± 0.0^b^ | 0.0 ± 0.0^b^ |
| **NDV16** | 0.0 ± 0.0^b^ | 0.0 ± 0.0^b^ | 0.0 ± 0.0^b^ | 0.0 ± 0.0^b^ |
| **NDV32** | 0.0 ± 0.0^b^ | 0.0 ± 0.0^b^ | 0.0 ± 0.0^b^ | 0.0 ± 0.0^b^ |
| **NDV64** | 0.0 ± 0.0^b^ | 0.0 ± 0.0^b^ | 0.0 ± 0.0^b^ | 0.0 ± 0.0^b^ |
| **CNDV8** | 0.0 ± 0.0^b^ | 0.0 ± 0.0^b^ | 1.0 ± 0.1^b^ | 2.2 ± 0.0^b^ |
| **CNDV16** | 4.0 ± 0.2^b^ | 2.3 ± 0.1^b^ | 1.0 ± 0.1^b^ | 3.3 ± 0.7^b^ |
| **CNDV32** | 3.7 ± 0.2^b^ | 4.1 ± 0.2^b^ | 3.3 ± 0.2^b^ | 3.3 ± 0.2^b^ |
| **CNDV64** | 1.5 ± 0.2^b^ | 0.5 ± 0.3^b^ | 1.6 ± 0.3^b^ | 2.3 ± 0.2^b^ |
| **CNDV8+T** | 1.7 ± 0.1^b^ | 0.8 ± 0.4^b^ | 1.3 ± 0.4^b^ | 0.0 ± 0.0^b^ |
| **CNDV16+T** | 3.1 ± 0.1^b^ | 1.8 ± 0.1^b^ | 0.6 ± 0.1^b^ | 0.0 ± 0.0^b^ |
| **CNDV32+T** | 1.3 ± 0.1^b^ | 1.2 ± 0.1^b^ | 1.3 ± 0.1^b^ | 0.0 ± 0.0^b^ |
| **CNDV64+T** | 0.0 ± 0.0^b^ | 0.6 ± 0.1^b^ | 1.5 ± 0.1^b^ | 3.0 ± 0.2^b^ |
