# Supplementary material for: Regression of solid breast tumours in mice by Newcastle disease virus is associated with production of apoptosis related-cytokines
Source: BMC Cancer. 2019 Apr 4;19:315. doi: 10.1186/s12885-019-5516-5 (PMC6449948; doi:10.1186/s12885-019-5516-5)
Supplement: Supplementary file 4 — Table S4. Concentration of IL-10 in both the NDV treated and control groups expressed in pg/ml throughout week 1 to week 4. (DOCX 15 kb) [file 12885_2019_5516_MOESM4_ESM.docx]

**Table S4:**

| **Groups/Week** | **Week 1** | **Week 2** | **Week 3** | **Week 4** |
| --- | --- | --- | --- | --- |
| **NC** | 0.1 ± 0.2 | 0.04 ± 0.1 | 0.0 ± 0.0 | 0.0 ± 0.0 |
| **CC** | 16.4 ± 0.3^a^ | 18.5 ± 3.0^a^ | 20.9 ± 2.2^a^ | 32.2 ± 2.1^a^ |
| **CT** | 0.0 ± 0.0^b^ | 0.0 ± 0.0^b^ | 0.0 ± 0.0^b^ | 0.0 ± 0.0^b^ |
| **NDV8** | 0.0 ± 0.0^b^ | 0.0 ± 0.0^b^ | 0.0 ± 0.0^b^ | 0.0 ± 0.0^b^ |
| **NDV16** | 0.0 ± 0.0^b^ | 0.0 ± 0.0^b^ | 0.0 ± 0.0^b^ | 0.0 ± 0.0^b^ |
| **NDV32** | 0.0 ± 0.0^b^ | 0.0 ± 0.0^b^ | 0.0 ± 0.0^b^ | 0.0 ± 0.0^b^ |
| **NDV64** | 0.0 ± 0.0^b^ | 0.0 ± 0.0^b^ | 0.0 ± 0.0^b^ | 0.0 ± 0.0^b^ |
| **CNDV8** | 4.3 ± 0.2^b^ | 7.2 ± 0.6^b^ | 10.2 ± 1.3^b^ | 13.2 ± 1.0^b^ |
| **CNDV16** | 17.1 ± 0.1^b^ | 13.1 ± 0.1^b^ | 0.0 ± 0.0^b^ | 0.0 ± 0.0^b^ |
| **CNDV32** | 29.5 ± 0.4^b^ | 30.1 ± 2.0^b^ | 32.1 ± 0.2^b^ | 30.4 ± 0.3^b^ |
| **CNDV64** | 12.1 ± 0.2^b^ | 14.3 ± 0.2^b^ | 23.4 ± 1.0^b^ | 25.4 ± 0.3^b^ |
| **CNDV8+T** | 0.0 ± 0.0^b^ | 0.0 ± 0.0^b^ | 0.0 ± 0.0^b^ | 2.4 ± 0.1^b^ |
| **CNDV16+T** | 11.3 ± 0.6^b^ | 5.3 ± 0.1^b^ | 0.7 ± 0.2^b^ | 0.0 ± 0.0^b^ |
| **CNDV32+T** | 0.0 ± 0.0^b^ | 0.6 ± 0.1^b^ | 2.3 ± 0.2^b^ | 0.0 ± 0.0^b^ |
| **CNDV64+T** | 0.0 ± 0.0^b^ | 0.7 ± 0.1^b^ | 0.7 ± 0.1^b^ | 0.0 ± 0.0^b^ |
