# Supplementary material for: Regression of solid breast tumours in mice by Newcastle disease virus is associated with production of apoptosis related-cytokines
Source: BMC Cancer. 2019 Apr 4;19:315. doi: 10.1186/s12885-019-5516-5 (PMC6449948; doi:10.1186/s12885-019-5516-5)
Supplement: Supplementary file 5 — Table S5. Concentration of IL-12p70 in both the NDV treated and control groups expressed in pg/ml throughout week 1 to week 4. (DOCX 15 kb) [file 12885_2019_5516_MOESM5_ESM.docx]

**Table S5:**

| **Groups/Week** | **Week 1** | **Week 2** | **Week 3** | **Week 4** |
| --- | --- | --- | --- | --- |
| **NC** | 8.5 ± 0.4 | 9.2 ± 0.3 | 7.3 ± 0.6 | 6.1 ± 0.5 |
| **CC** | 25.8 ± 1.0^a^ | 22.6 ± 10.7^a^ | 18.6 ± 3.0^a^ | 17.4 ± 5.2^a^ |
| **CT** | 21.5 ± 0.5^b^ | 23.5 ± 0.6 | 27.2 ± 3.6^b^ | 34.1 ± 0.2^b^ |
| **NDV8** | 8.3 ± 0.3^b^ | 7.9 ± 0.6^b^ | 8.3 ± 0.5^b^ | 8.0 ± 0.1^b^ |
| **NDV16** | 13.3 ± 0.6^b^ | 13.3 ± 0.2^b^ | 13.4 ± 0.2^b^ | 12.9 ± 0.2^b^ |
| **NDV32** | 14.6 ± 0.3^b^ | 13.1 ^b^ ± 2.3^b^ | 11.3 ± 0.1^b^ | 12.4 ± 0.1^b^ |
| **NDV64** | 4.9 ± 0.1^b^ | 12.9 ± 0.2^b^ | 10.4 ± 0.6^b^ | 10.2 ± 0.3^b^ |
| **CNDV8** | 12.0 ± 0.1^b^ | 11.3 ± 1.7^b^ | 10.5 ± 0.5^b^ | 5.5 ± 0.2^b^ |
| **CNDV16** | 18.0 ± 0.9^b^ | 13.3 ± 1.3^b^ | 11.5 ± 0.5^b^ | 8.5 ± 0.4^b^ |
| **CNDV32** | 15.1 ± 0.1^b^ | 13.2 ± 1.2^b^ | 11.6 ± 0.1^b^ | 9.0 ± 0.1^b^ |
| **CNDV64** | 5.0 ± 0.1^b^ | 12.3 ± 0.1^b^ | 18.3 ± 0.2 | 17.6 ± 0.3 |
| **CNDV8+T** | 10.7 ± 0.2^b^ | 10.8 ± 0.9^b^ | 10.3 ± 1.2^b^ | 10.0 ± 0.1^b^ |
| **CNDV16+T** | 7.3 ± 0.2^b^ | 15.5 ± 2.7^b^ | 25.3 ± 1.1^b^ | 23.7 ± 0.9^b^ |
| **CNDV32+T** | 12.8 ± 0.1^b^ | 37.7 ± 0.6^b^ | 50.7 ± 2.9^b^ | 33.4 ± 0.8^b^ |
| **CNDV64+T** | 30.1 ± 1.9 ^b^ | 27.4 ± 1.2 | 21.1 ± 1.0 | 15.2 ± 0.8 |
