# Supplementary material for: Regression of solid breast tumours in mice by Newcastle disease virus is associated with production of apoptosis related-cytokines
Source: BMC Cancer. 2019 Apr 4;19:315. doi: 10.1186/s12885-019-5516-5 (PMC6449948; doi:10.1186/s12885-019-5516-5)
Supplement: Supplementary file 6 — Table S6. Concentration of TNF-α in both the NDV treated and control groups expressed in pg/ml throughout week 1 to week 4. (DOCX 15 kb) [file 12885_2019_5516_MOESM6_ESM.docx]

**Table S6:**

| **Groups/Week** | **Week 1** | **Week 2** | **Week 3** | **Week 4** |
| --- | --- | --- | --- | --- |
| **NC** | 1.2 ± 0.2 | 2.5 ± 0.2 | 2.1 ± 0.1 | 3.0 ± 0.3 |
| **CC** | 5.6 ± 0.5^a^ | 3.7 ± 1.1^a^ | 18.6 ± 2.5^a^ | 4.2 ± 1.8^a^ |
| **CT** | 2.4 ± 0.4^b^ | 2.7 ± 0.1 | 1.4 ± 0.4^b^ | 3.1 ± 0.3^b^ |
| **NDV8** | 2.9 ± 0.6^b^ | 2.6 ± 0.2 | 8.3 ± 0.4^b^ | 8.0 ± 0.3^b^ |
| **NDV16** | 6.1 ± 0.1^b^ | 5.6 ± 0.3^b^ | 3.4 ± 0.3^b^ | 12.9 ± 0.1^b^ |
| **NDV32** | 3.5 ± 0.3 | 3.7 ± 0.2 | 3.9 ± 0.1^b^ | 3.7 ± 0.2 |
| **NDV64** | 11.9 ± 0.6^b^ | 5.1 ± 0.1^b^ | 1.7 ± 0.4^b^ | 2.9 ± 0.1^b^ |
| **CNDV8** | 2.2 ± 0.1^b^ | 2.1 ± 0.2^b^ | 2.4 ± 0.2^b^ | 2.4 ± 0.1^b^ |
| **CNDV16** | 16.3 ± 0.2^b^ | 13.3 ± 0.1^b^ | 2.4 ± 0.4^b^ | 3.7 ± 0.4 |
| **CNDV32** | 5.3 ± 0.2 | 4.6 ± 1.1 | 4.2 ± 0.2^b^ | 4.4 ± 0.2 |
| **CNDV64** | 2.6 ± 0.3^b^ | 3.6 ± 0.4 | 6.3 ± 0.1^b^ | 5.4 ± 0.2^b^ |
| **CNDV8+T** | 3.5 ± 0.2^b^ | 2.6 ± 0.1 | 2.0 ± 0.1^b^ | 1.7 ± 0.1^b^ |
| **CNDV16+T** | 2.4 ± 0.2^b^ | 2.4 ± 0.2^b^ | 1.9 ± 0.1^b^ | 0.5 ± 0.1^b^ |
| **CNDV32+T** | 3.4 ± 0.2^b^ | 3.5 ± 0.3 | 2.4 ± 0.1^b^ | 1.9 ± 0.1^b^ |
| **CNDV64+T** | 2.6 ± 0.3^b^ | 4.1 ± 2.0 | 4.3 ± 0.1^b^ | 5.1 ± 0.1^b^ |
